# The relationship between sales of SSRI, TCA and suicide rates in the Nordic countries

**DOI:** 10.1186/1471-244X-10-62

**Published:** 2010-08-06

**Authors:** Per-Henrik Zahl, Diego De Leo, Øivind Ekeberg, Heidi Hjelmeland, Gudrun Dieserud

**Affiliations:** 1 Department of Suicide Research and Prevention, Division of Mental Health, Norwegian Institute of Public Health, Oslo, Norway; 2 Australian Institute for Suicide Research and Prevention, Griffith University, Mt Gravatt Campus, Brisbane, QLD, Australia; 3 Department of Acute Medicine, Ullevaal University Hospital, Oslo, and Department of Behavioural Sciences in Medicine, Institute of Basic Medical Sciences. Faculty of Medicine, University of Oslo, Norway; 4 Department of Social Work and Health Science, Norwegian University of Science and Technology, Trondheim, Norway

## Abstract

**Background:**

In the period 1990-2006, strong and almost equivalent increases in sales figures of selective serotonin re-uptake inhibitors (SSRIs) were observed in all Nordic countries. The sales figures of tricyclic antidepressants (TCAs) dropped in Norway and Sweden in the nineties. After 2000, sales figures of TCAs have been almost constant in all Nordic countries. The potentially toxic effect of TCAs in overdose was an important reason for replacing TCAs with SSRIs when treating depression. We studied whether the rapid increase in sales of SSRIs and the corresponding decline in TCAs in the period 1990-98 were associated with a decline in suicide rates.

**Methods:**

Aggregated suicide rates for the period 1975-2006 in four Nordic countries (Denmark, Finland, Norway and Sweden) were obtained from the national causes-of-death registries. The sales figures of antidepressants were provided from the wholesale registers in each of the Nordic countries. Data were analysed using Fisher's exact test and Pearson's correlation coefficient.

**Results:**

There was no statistical association (P = 1.0) between the increase of sales figures of SSRIs and the decline in suicide rates. There was no statistical association (P = 1.0) between the decrease in the sale figures of TCAs and change in suicide rates either.

**Conclusions:**

We found no evidence for the rapid increase in use of SSRIs and the corresponding decline in sales of TCAs being associated with a decline in the suicide rates in the Nordic countries in the period 1990-98. We did not find any inverse relationship between the increase in sales of SSRIs and declining suicide rates in four Nordic countries.

## Background

In many Western countries, including Norway, Sweden, Denmark and Finland, suicide rates have been declining since the end of the 1980s [1,2]. The decline in Norway and Sweden, from the end of the eighties onwards, has been followed by relative stability over the last decade. Over the same time period, the sales figures of antidepressants have increased in all the Nordic countries. Selective serotonin re-uptake inhibitors (SSRIs) were introduced to the market around 1990. The sales figures of these drugs have subsequently increased year by year, while the sales figures of the potentially toxic tricyclic antidepressants (TCAs) - drugs which can be quite toxic in overdose - have been significantly reduced. Today, SSRIs constitute about two-thirds of the total sales figures of antidepressants in the Nordic countries [2].

To explain the reduction in the suicide rates seen in many countries over the last decades, a number of researchers have argued in support of an overall inverse relation between sales of antidepressants and suicide rates. For example, in a study published in 2000 by Isacsson in Sweden, the researcher argued that increased use of antidepressants "appeared to be one of the contributing factors to the decrease in the suicide rate". In a recent study on the sales of antidepressants and suicide rates in Norway, the authors stated that the fall in suicide rates was related to the increased sales of non-tricyclic antidepressants, but only for the lower sales segment, indicating that the switch from the more toxic TCAs could explain the observed relationship [3].

In the Nordic countries, the suicide rates started falling before the SSRIs were introduced [2]. In England and Wales, a declining trend coincided with the introduction of SSRIs for young men, while the suicide rates have been declining for three decades for young women [4]. Some have been concerned that SSRIs may even increase suicide risk in young people; however, there is no evidence of an overall effect of regulatory actions to restrict prescribing SSRIs to young people internationally [5].

Several published studies in favour of an inverse relationship between sales or use of antidepressants and suicide rates were performed utilizing an ecological research design. Some investigators have suggested that the relationship was causal [6-10]. In contrast, a recent review article based on publication presenting data on annual rates of completed suicide in relation to annual rates of SSRI prescription/use over the past decades, Safer and Zito concluded that "...available ecological evidence does not support an inverse temporal relationship between rates of completed suicide and SSRI utilization" [[11], p. 274], and "There is no consistent ecological pattern of completed suicide in relation to SSRI usage" [[11], p. 276]. Consistency is one of the Bradford Hill's criteria of causation [12].

Since the early nineties, suicide prevention has been high on the political agenda in the Western world. Medical treatment of depression is a main strategy for prevention, and detoxification of substances has been an important element in suicide prevention programs [13]. Thus, a reduction of suicide rates related to intoxication from antidepressants may be expected when a large number of people switch from using TCAs to using SSRIs [3], similar to what happened when doctors stopped prescribing barbiturates [14].

The aim of the paper is to study the relationship between increased sales of SSRIs and changes in the suicide rates in four Nordic countries in the time period 1990-98. A secondary aim is to study if the rapid switch from TCAs to SSRIs in the early nineties had any impact on the suicide rates in the same countries.

## Methods

The suicide rates for the period 1975-2006 in four Nordic countries (Denmark, Finland, Norway and Sweden) were obtained from the national causes-of-death registries. In the period 1975-85, the causes of death were classified using the ICD (International Classification of Diseases) 8th revision; in 1986-95 ICD-9 was used (not in Denmark), and from 1996 the causes of deaths were classified using the ICD-10 system. In the period 1991-2006 there were 13,401 suicides in Denmark, 19,781 in Finland, 8,821 in Norway and 19,975 in Sweden.

**Figure 1:**
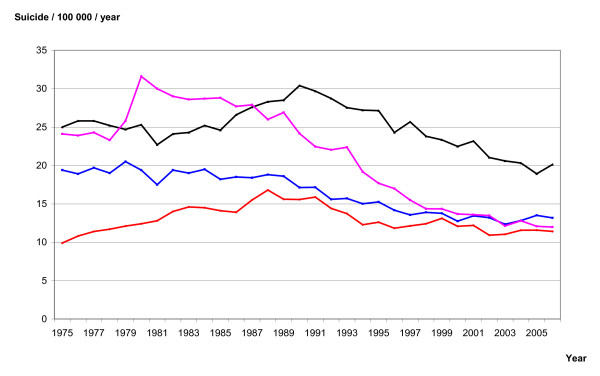
**Suicide rates in Denmark (violet), Finland (black), Norway (red) and Sweden (blue) in the period 1975 to 2006**.

Altogether the statistical material covers 61,978 suicides. In the previous period, 1975-90, about 65,500 suicides were recorded. National suicide rates are presented as crude rates (suicides per 100,000 individuals) in Figure 1.

**Figure 2:**
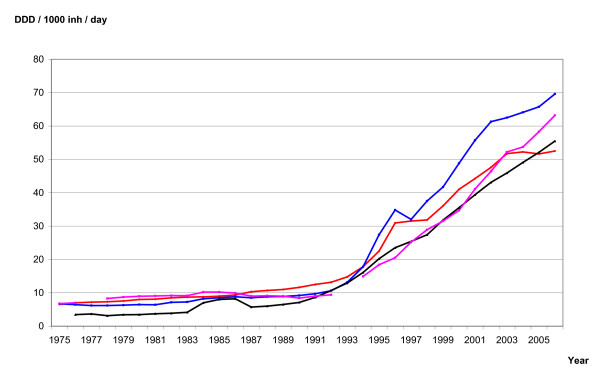
**Total sale figures of antidepressants (N06A) in Denmark (violet), Finland (black), Norway (red) and Sweden (blue)**.

**Figure 3:**
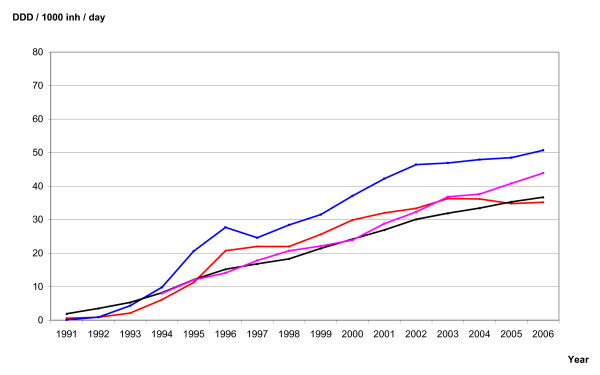
**Sale figures of SSRIs (N06AB) in Denmark (violet), Finland (black), Norway (red) and Sweden (blue)**.

**Figure 4:**
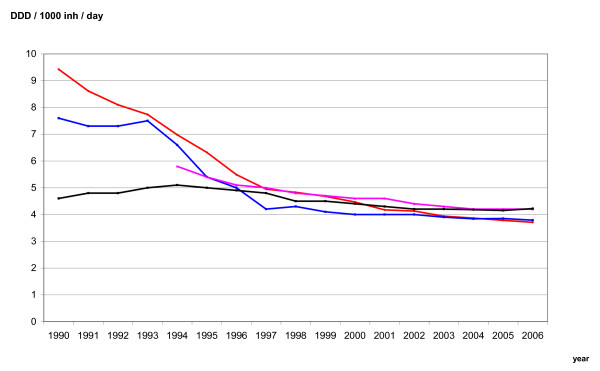
**National sale figures of TCA (N06AA) in Denmark (violet), Finland (black), Norway (red) and Sweden (blue)**.

The sales figures of antidepressants were provided from the wholesale registers in each of the Nordic countries. These data represent total sales to pharmacies and institutions. Even though the data are complete, they do not necessarily represent consumption because not all drugs being sold are consumed. In the Nordic countries, drugs on the market are grouped according to the Anatomical Therapeutic Chemical (ATC) classification. The total national sales figures of ATC group N06A (all antidepressants), N06AB (SSRIs) and N06AA (TCAs) were recorded, and the sales figures were standardised as defined daily doses (DDD)/1,000 inhabitants/day for the period 1975-2006. The sales figures are standardized as DDD/1,000 inhabitants/day, and presented graphically for all antidepressants (Figure 2), SSRIs (Figure 3) and TCAs (Figure 4). Note that Danish sales figures are missing in the period 1990-93.

To investigate the association between SSRIs, TCAs and suicide rates, we have restricted the statistical analyses to the period 1990-98 where changes in the SSRI and TCA sales figures were largest and when one would expect the largest fall in the suicide rates. We used a non-parametric method to study the associations. First we calculated the annual differences (the difference between numbers in two succeeding years) in the suicide rates and the sales figures of SSRIs and TCAs, respectively. We calculated eight differences for Finland, Norway, and Sweden but 4 differences for Denmark because sales figures were missing for 1990-93. We studied whether years with large increases in sale figures were associated with large drops in the suicide rates using Fisher's exact test [15] to calculate the significance values for the 2 × 2 tables. Our null hypothesis was that there is no association. The alternative is that there was either a strong negative or positive association. The latter, we believe, is not medically plausible.

First, we ranked the 28 annual differences in suicide rates into four quartiles; there are seven differences in each quartile (first column in Table 1). The corresponding differences in the SSRI sales figures are presented (second column in Table 1). Then we identified all SSRI sales figure differences under the median difference. Under the null hypothesis (there is no association between the sales figures of SSRIs and the suicides) a similar number of SSRI differences below as well as above the median SSRI difference should have been observed in each quartile of suicide differences.

**Table 1:** Ranked annual changes in suicide rates (first column), annual changes in the sales figures of SSRIs (second column) and annual changes in the sales figures of TCAs (third column) in the period 1990-98.

Differences in suicide rates	Differences in sale figures of SSRI	Differences in sale figures of TCA	Country
-2.85	0.59 *	-0.1	3
-1.88	0.29 *	-0.3 *	3
-1.57	1.24 *	0	2
-1.5	3.99	-0.1	4
-1.49	5.17	-0.4 *	4
-1.47	9.46	-0.51*	1
-1.45	1.25 *	-0.76 *	1

-1.19	-0.03 *	0.2	3
-1.13	0.10 *	-0.2	4
-1.09	0.80 *	-0.4 *	2
-0.96	3.40	0	3
-0.77	5.50	-0.83 *	1
-0.72	10.80	0.2	3
-0.69	7.10	-0.36 *	1

-0.69	-3.10 *	-0.3	4
-0.68	3.80	-0.9 *	2
-0.6	1.90 *	-0.8 *	2
-0.33	1.60 *	0.1	3
-0.06	1.80 *	-0.1	3
0.03	2.90	-0.3 *	2
0.11	3.90	0.2	2

0.24	3.10	-1.2 *	2
0.27	1.60 *	-0.12	1
0.3	1.50 *	-0.54 *	1
0.32	4.00	-0.81 *	1
0.32	2.10 *	-0.66 *	1
0.32	3.70	0.1	2
1.39	2.90	-0.1	3

In the fourth column, we have Norway (1), Sweden (2), Finland (3) and Denmark (4).

Differences in sales figures below the median are marked with a star.

We also used the same method to study whether decreases in the sales figures of TCAs (third column in Table 1) were associated with a decline in suicides rates. This second statistical analysis is supplementary to the analysis of the relationship between SSRIs and suicide rates, but also an independent test of a possible relationship between sales of TCAs and suicide rates.

It is also possible to look at the distributions of SSRIs and TCAs sales figures differences within each quartile. When there are 3 or 4 differences under the median in each quartile, there is no significant association. One may study all four quartiles simultaneously, or alternatively, one can study first and forth quartile separately.

Finally we estimated the Pearson correlation coefficients for the differences, and we also studied how much of the observed drop in suicide rates was actually explained by a drop in intoxications.

## Results

The Nordic suicide rates for the period 1975-2006 are presented in Figure 1. Finland had the highest suicide rates throughout the period. The suicide rates in Finland, Norway, Sweden and Denmark have been declining after 1990. After 1999 there was a marginal, but not significant fall in the suicide rates in Norway, Sweden and Denmark (P = 0.055 when testing for a trend using a Poisson regression model adjusting for country differences. Trends are still not significant when using an autoregressive model for each country). The previous difference between Norway, Sweden and Denmark is almost eliminated, while Finland still has a higher rate compared to the other three countries.

The total sales figures of antidepressants increased by 51 percent during the period 1975-1989 in the four Nordic countries (Figure 2). In the period 1975-1989, Finland had the lowest sales figures (the average sales number was 4.7 DDD/1,000 inhabitants/day), while Denmark had the highest ones (the average sale was 8.5 DDD/1,000 inhabitants/day). Then, from the early 1990s on to 2006, the sales figures started to increase from 10 to more than 50 in all four countries.

The national sales figures of SSRIs are presented in Figure 3. The sales figures are constantly increasing in the period 1991-2006 for all countries except Norway, where the increase leveled out after 2004. In 2006, sales of SSRIs constituted 67 percent of the total sales of antidepressants in Norway, 73 percent in Sweden, 66 percent in Finland and 69 percent in Denmark. About one-third of the increase in SSRI sales figures is after 2000 when suicide rates are constant.

The sales figures of the TCAs in the period 1990-2006 are presented in Figure 4. Sales figures of TCAs in Denmark in the period 1990-93 were not available. However, total sales figures for antidepressants in Denmark in these years were 8.5, 9.0 and 9.4, respectively. Assuming the same percentage level of TCAs in Denmark as in the other countries, compared to the total sales figures for antidepressants, the sales figures of TCAs would be about 7.0 in 1990-93. In 1990, the TCAs constituted 65 percent of all antidepressants in Finland, and about 80 percent in the other three countries. In 2006, the TCAs constituted 5-8 percent of all antidepressants; however, the absolute fall from 1990 to 2006 was only about 50 percent.

In Table 1, the 28 differences in the suicide rates for the four countries in the period 1990-98 are ranked in the first column. The horizontal lines split the ranked mortality differences into four quartiles with seven differences in each. There are 14 differences in suicide rates above the median (upper 14) and 14 differences below the median (lower 14). The median suicide difference is -0.69. Negative differences indicate that the suicide rate declined and positive differences indicate that the suicide rate increased. In the second column, the corresponding changes in the sales figures of SSRIs are presented. These are not ranked (as the differences in the first column), but the numbers marked with a star are the differences in the sales figures below the median differences in the sales figures (the median is 2.5).

In Table 2, we present the 2 × 2 table for all outcome variables; among the 14 lowest suicide differences (from -2.85 to -0.69), there are seven SSRI differences under the median SSRI difference and seven above; among the14 highest differences (from -0.69 to 1.39) there are also seven SSRI differences under the median SSRI difference and seven above. The P-value in the exact Fisher test is 1.0. The Pearson correlation coefficient between differences in SSRI and suicide rates is 0.06 (P = 0.76). We also conducted a subgroup analysis of sales figures differences in the first quartile (the seven largest drops in the suicide rates) and fourth quartile (seven largest increases in differences). The numbers of cases in each cell are three or four, and the P-value is 1.0 when using the Fisher test statistics.

**Table 2:** Numbers of SSRI differences and suicide rate differences below and above the median.

	SSRI differences below median	SSRI differences above median
Suicide rates below median	7	7
Suicide rates above median	7	7

The changes in the sales figures of TCAs are presented in the third column in Table 1.

The Pearson correlation coefficient between TCAs and SSRIs differences is -0.36. The numbers marked with a star are once again the differences in the TCAs sales figures below the median difference (the median difference is -0.3). Here, the Pearson correlation coefficient between differences in TCAs and suicide rates is -0.10 (P = 0.76). In Table 3, the 2 × 2 table for all suicides and TCAs differences is presented; the 28 differences are equally distributed in all cells (as above) and the P-values for the Fisher test is 1.0. We also considered whether there was a lagged response on the suicide rates. We studied the associations between changes in TCAs sales figures in one year and the changes in the suicide rates in the following year. However, this had no effect on the results. Additionally, we looked at the association for only the data in the first quartile (the seven largest drops in the suicide rates) and the fourth quartile (the seven largest increases in the suicide rate). The number of cases in each cell is three or four, and the P-value is 1.0 when using the Fisher test statistics.

**Table 3:** Numbers of TCA differences and suicide rate differences below and above the median.

	TCA differences below median	TCA differences above median
Suicide rates below median	7	7
Suicide rates above median	7	7

Finally, we studied the proportion of suicides in Norway caused by intoxication in the periods 1991-95 and 1996-2000. There were 2,960 and 2,725 suicides, respectively. The numbers of suicides caused by intoxication were 735 and 605, respectively. The drop in intoxication was 130, while the drop in suicides was 235. Thus, almost half of the drop in Norway is not explained by the drop in intoxications, which is in accordance with our statistical analyses.

## Discussion

Two issues were investigated in the present study: 1) whether there is a statistical inverse relationship between the sales of SSRIs and the suicide rates in four Nordic countries in the time period 1990-98, and 2) whether the switch from TCAs to SSRIs has had a significant impact on the suicide rate.

We found no negative association between the increasing sales figures of SSRIs and the declining suicide rates in the period using our study design. We did not find any association between the rapid decline in the sales figures of TCAs and the simultaneous decline in the suicide rates either.

The sales figures of antidepressant were slowly increasing in all four countries before 1990 (Figure 2). In contrast, the corresponding suicide rates differed between the nations, increasing in Norway and Finland, and slowly decreasing in Sweden (Figure 1). After 1999, sales figures of SSRIs are still increasing in Norway, Sweden and Denmark, but there is no significant decline in the corresponding suicide rates. Thus, there is no consistency in the associations between the trends in figures 1 and 2 before 1990. Consistency is one of the Hill's criteria of causation [12].

We wanted to see if a strong increase in sales figures of SSRI in one year was associated with a strong decline in suicide rates in the same year; i.e. if there was a dose-response relationship. Our analysis is inspired by the dose-response causal criteria [12]. The analyses in Tables 2-3 are simplified versions of the Mantel-Haenszel method [16], which is commonly used to study dose-response relationships. Stratifying data into quartiles instead of two groups also gave non-significant results.

The associations between the curves in figures 1 and 2 can also be studied using alternative statistical methods. Correlation coefficients estimate the strength of a linear association between two variables. Regression models allow for adjustment of trends and confounding. Our method does not assume linear relationships and we do not have to model the dose-response relationship either.

Because intoxication only accounts for about 25 percent of all suicides and because intoxications are caused by many other substances than TCAs, a simple calculation shows that a 50 percent decline in the absolute sales figures of TCAs can only have a marginal effect on the overall suicide rates, if there is any at all. For example, if 20 percent of all fatal intoxications are caused by TCAs in Norway, then the predicted reduction in the suicide rate is (20-10)/4% = 2.5%. This means that the observed reduction in TCA sales figures can only explain a small part of the observed reduction in suicide rates.

The two hypotheses above are related because many people have switched from using TCAs to using SSRIs. Because the sales figures of TCAs and SSRIs are negatively correlated, one may argue that the statistical analyses are mathematically dependent. However, the correlation coefficient is only -0.36, and of fourteen TCA differences below the TCA median difference, only six of the corresponding SSRI differences were above the median SSRI differences.

During the period 1990-98 the sales figures of SSRIs increased more than the sales figures of TCAs declined, allowing more people to be treated; however, there is no association between increased sales of SSRIs and decrease in the suicide rates. In contrast, Bramness et al. [3] reported a small time-dependent inverse association in Norway, but when including data from the other Nordic countries, this association disappears. If increased use of antidepressants (and more patients being treated) prevents suicide, this will cause an underlying declining trend in suicide rates.

Our study of the relationship between sales of SSRIs and suicide rates is using the same data as Reseland et al. [2]; however, their conclusion is based on when the suicide rates started to decline and not on the direct association between sales figures and suicide rates per se. The decline in the suicide rates in Denmark and Sweden pre-dated the introduction of SSRIs by ten years or more, and the rates continued to decline thereafter [2]. In Norway, the association was only present in the first three years after the introduction of SSRIs, and during the period when the major increase in sales of SSRIs occurred, there were no major changes in the suicide rates [2,3]. The estimated association reported in Norway [3] is only valid for a small subset (less than 10 percent) of the Norwegian data. In Finland, an association between increased sales of antidepressants and reduction of suicide rates has been demonstrated, but the effect was not as strong for females as for males [2]. In Iceland, the sales figures are the highest in the Nordic Countries, but suicide rates have been relatively constant for 50 years [17]. Thus, there is mixed evidence that increased use of antidepressants has coincided with a reduction in suicides [2].

The association between the decline in suicide rates in Norway and the increase in sales figures of SSRIs is different from the other Nordic countries. Initially, the association is very strong but after three years (post 1994) there is no association. Thus, the conclusion by Bramness and colleagues [3], that "the fall in suicide rates in Norway and its counties was related to the increased sales of non-TCAs" seems not to be warranted. The authors claim that the effect was mostly a result of a sales increase in the lower sales segment (page 1), due to "a change from the more toxic TCAs, or heightened awareness of depression and its treatment " (page 1). However, in Denmark there was no inverse association between sales of SSRIs and suicide rates in the same period, while there was such an association after 1994. The same pattern was found in Sweden and Finland, while there was no inverse association in Norway after 1994. Further, in all Nordic countries, except Norway, associations are also present in the higher sales segment. Thus, in the present study, we have shown that the claimed association in Norway [3] is contradicted when using an alternative analysis and including data from all Nordic countries. In the US, an inverse association between the suicide rate and the use of SSRIs has been demonstrated for the time period 1990-2000, but more detailed analyses have revealed that the association is only valid for older and male adolescents residing in low-income regions. Furthermore, the analyses did not include factors like psycho-social interventions and substance abuse [18]. In Australia, Hall et al. [7] claimed a link between increased use of SSRIs and reduced rates of suicide. Their study attracted a number of reactions, pointing at a simple shift in prescribing full-dose SSRIs instead of low-dose TCAs [19] and a marked pre-existing decline in elderly suicide rates [20], essentially related to the strong reduction in overdosing on barbiturates as a suicide method [21]. In the words of Sakinofsky [22], "Hall's conclusion [...] overstates the evidence" (page 71).

The shift from TCAs to SSRIs in Denmark was similar to that in Norway [23]. The proportions of TCAs in the total sales figures of antidepressants in Norway and Denmark in 2001 were 9.5% and 11%, respectively. The proportion of TCAs in the total sales in Norway in 1991 was 69%. We do not know the proportion in Denmark; however, before 1983, proportions were similar between Norway and Denmark [23]. The inconsistencies between the patterns in different countries are also shown by the different trends in Norway and Sweden during the period 1974-1988. The sales figures for antidepressants were rather similar in the two countries, and slightly increasing in both cases. During the same years, suicide rates in Norway increased by approximately 70%, whereas they decreased slightly in Sweden. The reduction in the incidence of suicides caused by intoxication in Norway can explain about half of the drop in suicides in the 1990s.

## Conclusions

This study was unable to find an association between rapid increase in use of SSRIs and corresponding decrease in sales of TCAs and the decline of suicide rates in four Nordic countries during 1990-98. In particular, no evidence was detected of an inverse relationship between the increase in sales of SSRIs and declining suicide rates.

In interpreting these findings, we need to keep in mind that there are factors in suicide phenomena that we still do not know [24]. Time concomitances in trends across nations and, sometimes, across cultures remain puzzling, especially when we try to understand why suicide rates have fallen both in countries that have and do not have a national suicide prevention strategy [1,25]. As far as we know, variables that are more difficult to measure than sales of antidepressants, such as cohort effects and other cultural influences, could theoretically have a bigger impact on suicide rates than any drug or psychotherapeutic/psychosocial treatment. Yet, at this stage, we are unable to convincingly comprehend suicide phenomena, including suicide trends. Combining different disciplines and competencies appears to be the most logical way of feeding hopes in overcoming the present difficulties [25-27]. We need to take into account that other suicide preventive measures have also been implemented since the early nineties, and might as well account for at least some of the reduction in suicide rates.

This study was restricted to national rates of suicide. It did not consider, as other investigations did [5,7], trends distinctively examined by gender and age groups. Furthermore, the study did not consider aspects such as under-prescription and under-treatment of depressed patients, their eventual compliance, and efficacy of antidepressants. A balanced review of current controversies in ecological studies on antidepressants and suicide can be found, for example, in Sakinofsky [22].

Lastly, the quality of the national suicide rates depends on the autopsy rates [28], which may vary over time and between countries. However, this problem may affect all kind of study designs, not only ours. Help-seeking and fear of stigmatization in patients (crucially important in effective therapies) were also left aside, as well as other factors able to influentially interact with suicide trends, such as alcohol consumption, illicit drugs use, and unemployment. With all these limitations kept in mind, we found no evidence that the rapid shift from using tricyclic antidepressants to using selective serotonin re-uptake inhibitors was associated with a decline in the suicide rates.

## Competing interests

The authors declare that they have no competing interests.

## Authors' contributions

PHZ, DDL and GD conceived the project. PHZ collected data and GD wrote the first draft. PHZ performed the statistical analyses. All authors have contributed to writing and revising the manuscript, and all authors read and approved the final manuscript.

## Pre-publication history

The pre-publication history for this paper can be accessed here:

http://www.biomedcentral.com/1471-244X/10/62/prepub
